# The Evolutionary Trend and Genomic Features of an Emerging Lineage of Elizabethkingia anophelis Strains in Taiwan

**DOI:** 10.1128/spectrum.01682-21

**Published:** 2022-01-19

**Authors:** Yu-Lin Lee, Kuan-Ming Liu, Hui-Lan Chang, Yi-Ci Liao, Jen-Shiou Lin, Fang-Yen Kung, Cheng-Mao Ho, Kai-Hsiang Lin, Ying-Tsong Chen

**Affiliations:** a Department of Internal Medicine, Changhua Christian Hospital, Changhua County, Taiwan; b Ph.D. Program in Medical Biotechnology, National Chung Hsing University, Taichung City, Taiwan; c Institute of Genomics and Bioinformatics, National Chung Hsing University, Taichung City, Taiwan; d Department of Laboratory Medicine, Changhua Christian Hospital, Changhua County, Taiwan; e Department of Clinical Pathology and Laboratory Medicine, Taichung Tzu Chi Hospital, Taichung City, Taiwan; f Biotechnology Center, National Chung Hsing University, Taichung City, Taiwan; g Institute of Molecular and Genomic Medicine, National Health Research Institutes, Miaoli County, Taiwan; Forschungszentrum Jülich GmbH

**Keywords:** *Elizabethkingia* species, multidrug resistance, nosocomial infection, comparative genomics

## Abstract

The incidence of Elizabethkingia anophelis bacteremia increased significantly in a tertiary hospital, Changhua Christian Hospital (CCH) since 2013. The infection density was 1.3 and 8.1 cases per 100,000 patient-days between 2005 and 2012 and 2013 and 2020, respectively (*P* < 0.05). During an outbreak investigation, a specific lineage of *E. anophelis* strains was identified by the pulsed-field gel electrophoresis analysis. To evaluate the evolution of the specific *E. anophelis* lineage, whole-genome sequencing was performed, and unique genomic features (GRs) were determined by comparative genomic analysis. The specific *E. anophelis* lineage was novel compared to worldwide strains ever reported by cg-MLST phylogenic and whole-genome comparative analysis. Multiplex PCR using primers designed from unique GRs were performed for prevalence screening among isolates from the CCH and nationwide isolates from the Taiwan surveillance of Antimicrobial Resistance (TSAR) Program. The proportion of the specific *E. anophelis* lineage increased from 7.9% (3/38) during 2005-2012 to 89.2% (223/250) during 2013-2020 (*P* < 0.05). Although *E. anophelis* usually confers resistance to multiple antibiotics with limited therapeutic options, the *E. anophelis* strains in the specific lineage had higher ciprofloxacin resistance (100% [226/226] versus 27.4% [17/62], *P* < 0.05) and was associated with a higher 14-day mortality rates (33.2% [37/226] versus 16.1% [10/62], *P* < 0.05) than other strains at CCH. A similarly increasing trend was also found in the national TSAR program during 2002-2018 (*p* for trend <0.05). We concluded that a novel lineage of *E. anophelis* strains has emerged dominantly in Taiwan. The genomic features are important for further investigations of epidemiology, resistance, virulence, and appropriate treatment.

**IMPORTANCE** Elizabethkingia anophelis is an emerging multidrug resistant pathogen caused several global outbreaks recently. *E. anophelis* was frequently misidentified as *E. meningoseptica* in the past by conventional culture methods; therefore, the prevalence was often underestimated. Through revised identification, an increasing trend of *E. anophelis* infection was noted in a tertiary hospital and a dominant lineage of strains was recognized by genotyping. To our best knowledge, the dominant lineage of *E. anophelis* is novel in comparison to other worldwide strains by whole-genome comparative analysis and several unique genomic regions were found. The whole-genome sequencing data also demonstrated multiple putative virulence factors and genes associated with multidrug resistance. In our study, we identified a specially evolved *E. anophelis* in Taiwan with increasing nationwide dominance. This study will assist in further epidemiology surveillance and developing corresponsive infection control policies to restrain it potential of global dissemination.

## INTRODUCTION

The genus *Elizabethkingia* is aerobic, nonfermenting, nonmotile, and non-spore-forming Gram-negative rods commonly found in the environment, particularly soil and water (1–3). *E. meningoseptica* is the first species in the genus reported to cause neonatal meningitis in 1959; however, growing evidence indicates that *E. anophelis* is the most prevalent pathogen in the genus associated with human infections (4–7). Since the first human case infected by *E. anophelis* in 2011 (8), an outbreak due to *E. anophelis* was reported in 2013 involving five patients in two intensive care units in Singapore (9). Another outbreak took place in the United States (US) between 2015 and 2016 (7, 10). During the investigation of the US outbreak, a unique *E. anophelis* strain was identified with a genomic feature of a disrupted DNA repair *mutY* gene (7). The disrupted *mutY* gene was associated with high evolutionary rate which might contribute to increased adaptability to environment (7). In Taiwan, the *E. anophelis* EM361-97 was sporadically discovered from the blood cultures of a cancer patient in 2017 and the phylogenetic analysis revealed *E. anophelis* EM361-97 was a sister group to *E. anophelis* FMS-007 in China (11).

*E. anophelis* has intrinsic multidrug resistance to a variety of antimicrobial classes, including penicillins, cephalosporins, β-lactam combination agents, carbapenems, and aminoglycosides. Besides, the susceptibility of *E. anophelis* to quinolone and trimethoprim-sulfamethoxazole are variable (12–15). Recently, doxycycline and minocycline were reported to inhibit more than 90% isolates in several studies with MICs less than 4 μg/ml (16–19). However, there is still controversial about the treatment regimen of *E. anophelis* infection.

Recently, we reported a 3-year outbreak of *E. anophelis* infection at a tertiary hospital in central Taiwan (20). A dominant lineage of *E. anophelis* strains was identified by pulsed-field gel electrophoresis (PFGE) analysis. In this study, we conducted whole-genome sequencing and comparative genomic analysis to identify genomic features of those *E. anophelis* strains and investigate its epidemiological trend.

## RESULTS

### The trends of incidence and antimicrobial resistance of *E. anophelis* infections.

**FIG 1:**
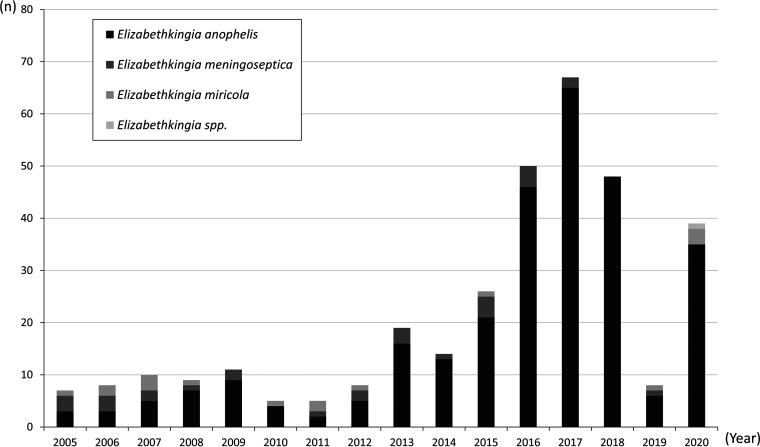
The distribution of different *Elizabethkingia* species in Changhua Christian Hospital from 2005 to 2020.

From 2005 to 2020, a total of 334 isolates of *Elizabethkingia* species were retrieved at CCH. After reidentification, *E. anophelis* was most dominant among all *Elizabethkingia* spp. (86.2%, 288/334), followed by *E. meningoseptica* (8.7%, 29/334), *E. miricola* (4.8%, 16/334), and *Elizabethkingia* spp. (0.3%, 1/334). A significantly increasing trend of *Elizabethkingia* species infection was noted with increases of the case number of *E. anophelis* more obviously than other two species since 2013 (Fig. 1). For *E. anophelis*, the infection density increased from 1.3 to 8.1 cases per 100,000 patient-days between 2005 and 2012 and 2013–2020, respectively (*P* < 0.05). The case number of *E. anophelis* bacteremia peaked in 2017 with 65 cases being identified in a year. The first intrahospital outbreak was detected in 2015 in a respiratory care center with 4 patients involved. A dominant lineage of *E. anophelis* strains had been identified according to PFGE analysis during the early outbreak investigations (20). Intensive infection control strategies were implemented since 2016 and the case number had been reduced gradually. Only 6 cases of *E. anophelis* bacteremia was recorded through the whole year in 2019. However, the incidence rebounded during the COVID-19 pandemics in 2020.

### The genomic features of the dominant lineage of *E. anophelis* strains.

**FIG 2:**
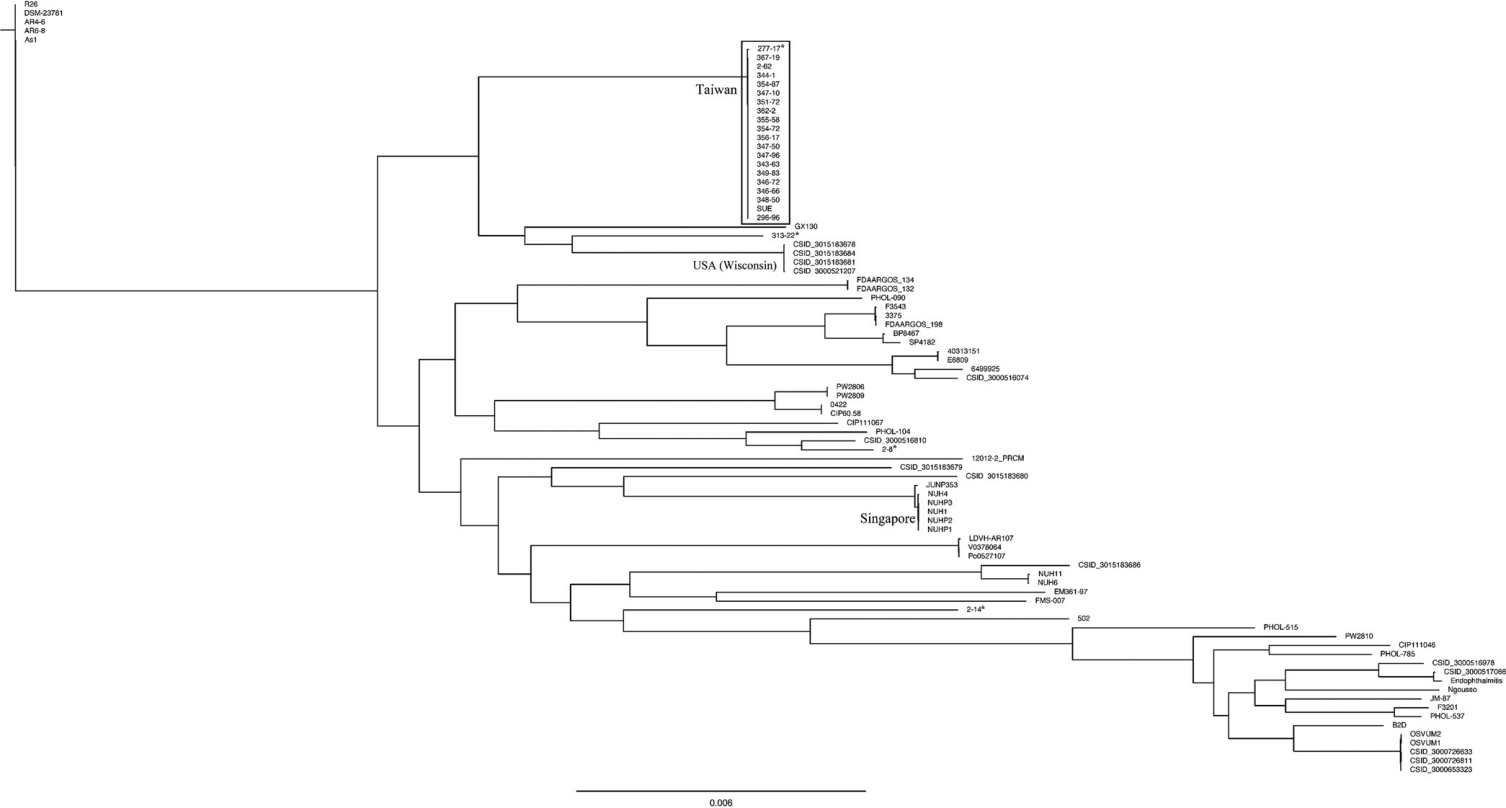
The phylogenetic tree constructed by core-genome multilocus sequence typing (cg-MLST) between 41 completed genomes (including 22 genomes in our study) and 45 draft genomes of Elizabethkingia anophelis. The 18 strains in the dominant lineage, *E. anophelis* 277-17, and SUE were in the same clade (rectangle). *, four strains not belonging to the dominant lineage according to PFGE analysis in our study, including *E. anophelis* 277-17, 313-22, 2-8, and 2–14.

Twenty-two *E. anophelis* strains included in previous PFGE studies were selected for complete genomic sequencing and comparative genomic analysis (20). The isolates, including 18 strains belonging to the specific lineage and 4 other *E. anophelis* strains were selected based on their pulsotypes. The sequencing data resulted in a single circular genome for each strain with the length ranging from 3,982,150 to 4,084,052 bp and the mean GC content ranging from 35.7% to 35.9%. The cg-MLST analysis was performed on 41 completed genomes and 45 draft genomes, including our 22 isolates and other worldwide strains. The details of all genomes used in cg-MLST analysis were summarized in Table S1. From about 4,000 genes in each of the genomes, 1,779 genes were selected as core genes to generate core genome scheme and multilocus alignment. In the cg-MLST phylogenetic tree, all 18 strains in the dominant lineage and one other *E. anophelis* isolate (strain 277-17) were within the same clade. These 19 isolates were very closely related to each other and formed a distinct clade which was separated from other isolates with a long branch (Fig. 2). Three of our *E. anophelis* strains (313-22, 2-8 and 2–14) were located outside the major clade.

**FIG 3:**
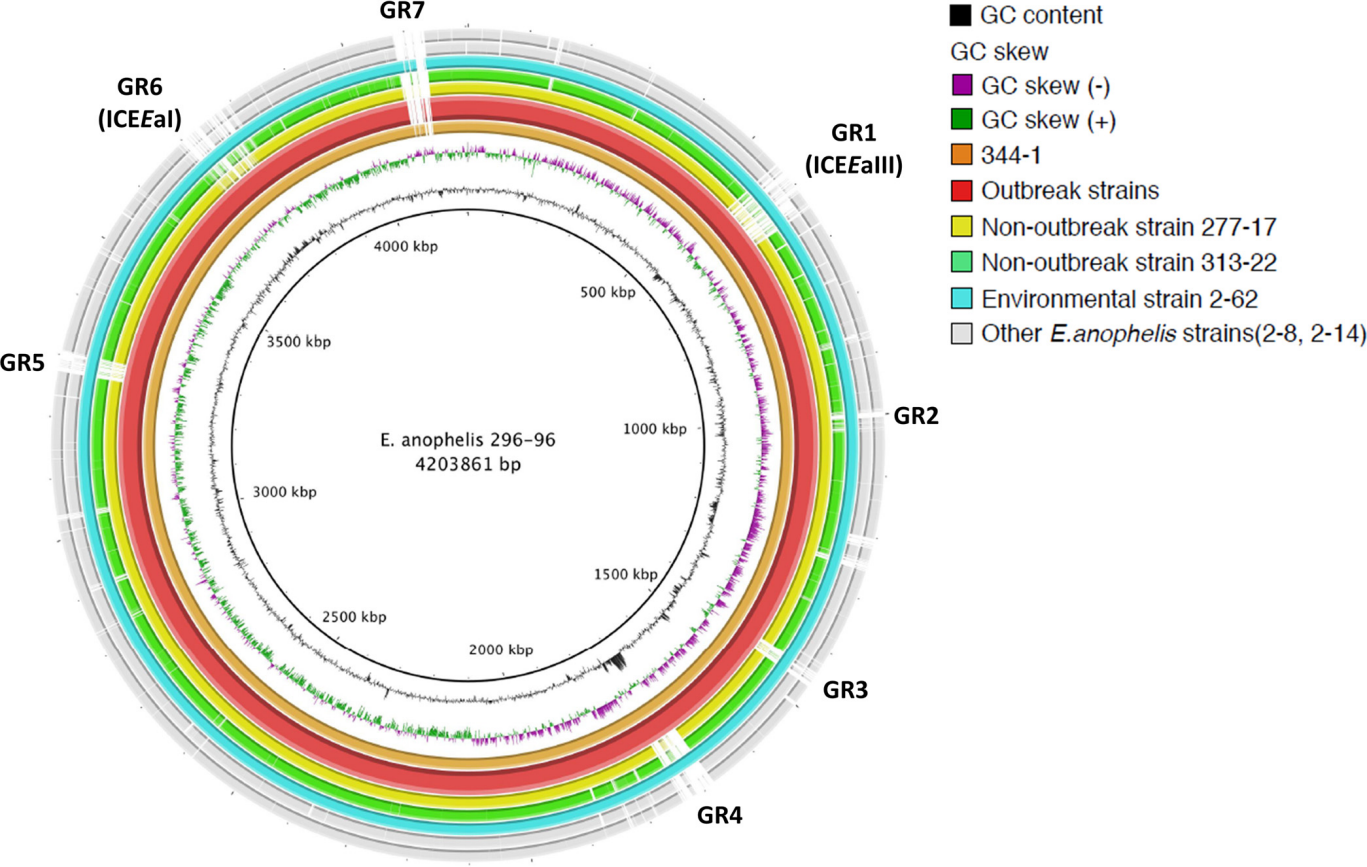
Genomic comparison among *Elizabethkingia* species. The genome of *E. anophelis* 296-96 (center) compared to *E. anophelis* 344-1 (the innermost circle; ring 1), outbreak strains, including 15 *E. anophelis* isolates (ring 2), non-outbreak strains, including *E. anophelis* 277-17 (ring 3) and *E. anophelis* 313-22 (ring 4), environmental strains, including *E. anophelis* 2–62 (ring 5) *E. anophelis* 2–8 (ring 6) and *E. anophelis* 2–14 (ring 7). Seven genomic regions (GRs) were identified different between outbreak and non-outbreak strains. GR1 and GR6 were type III and type I integrative and conjugative elements (ICEs), respectively.

For further comparative genomic analysis, we used *E. anophelis* 296-96 as a representative strain as it was the earliest strain in the dominant lineage. The results are illustrated with the use of the BLAST Ring Image Generator (BRIG) in Fig. 3. In comparison with strain 296-96, several genomic regions (GRs) ranging from 35-kb to 90-kb were found unique among strains in the dominant lineage. Those GRs were numbered from GR1 to GR7. All 18 strains in the specific lineage had GR1 to GR6, but GR7 was only present in strains 2–62, 344-1 and 296-96. The annotated genes in the seven GRs are listed in Table 1. The seven GRs were searched among all *E. anophelis* strains with available genome data in NCBI database to date by the Basic Local Alignment Search Tool (BLAST). The BLAST results disclosed no other isolates had similar GRs to our novel *E. anophelis* strains. The only exception was *E. anophelis* SUE, an isolate recently recovered from another hospital in central Taiwan (21). The genome of *E. anophelis* SUE had all six GRs and its genome was identical to those of our strains in the dominant lineage (Fig. S1). Analysis of the *E. anophelis* 296-96 genome using Islandviewer4, which is empowered by integrated genomic island predictors, have shown that 5 (GR1, 3, 4, 6, and 7) out of the 7 GRs are likely genomic islands. GR1 and GR3 were predicted positive, respectively, by SIGI-HMM and IslandPath-DIMOB. GR4, GR6 and GR7 were positive by both predictors (Table S2). Annotation using PHAST shows that a 47-kb region (4,081,340-4,127,081) located within GR7 is phage-related.

**TABLE 1:** Characteristics of seven genomic regions (GRs) presented among dominant Elizabethkingia anophelis strains but not among nondominant strains

Genomic region	Start	End	Size (nt)	Features
GR1	555,248	645,046	89,799	ICE*Ea*III; Mobile element protein, putative outer membrane protein, Single-stranded DNA-binding protein, DNA topoisomerase III (EC 5.99.1.2), RNA-directed DNA polymerase (Reverse transcriptase), Phage protein, Peptidase, M23/M37 family
GR2	989,660	1,027,265	37,606	Prolyl oligopeptidase family serine peptidase, MFS transporter, porin, response regulator, alkyl hydroperoxide reductase, peptidase M61, insulinase family protein, IS*3* family transposase
GR3	1,437,987	1,477,787	39,801	Mobilization protein *BmpH*, IS*Sod13*, transposase, CMP/dCMP deaminase, zinc-binding
GR4	1,679,590	1,741,370	61,781	Type II, N-methyl DNA methyltransferase (group alpha), T4CP, conjugative transposon protein Tra(G, J, K, M, N), Two-component transcriptional response regulator-*LuxR* family, RND efflux system, prophage pi2 protein 34
GR5	3,273,455	3,308,711	35,257	N-acetylmuramoyl-l-alanine amidase, Phage protein, RNA-directed DNA polymerase (Reverse transcriptase)
GR6	3,689,204	3,779,399	90,196	ICE*Ea*I; Tetracycline resistance element mobilization regulatory protein *RteC*, DNA topoisomerase III, Bacteroidales-type, Putative DNA methylase, TonB-dependent receptor; Outer membrane receptor for ferrienterochelin and colicins, Mn-dependent transcriptional regulator *MntR*, Alpha-acetolactate decarboxylase, Manganese ABC transporter, UDP-2,3-diacylglucosamine diphosphatase, Phosphoglycerate mutase, Methionine aminopeptidase, Ribonucleotide reductase of class Ia (aerobic), Peptide deformylase, Cytosol aminopeptidase *PepA*, Alkyl hydroperoxide reductase protein C, Superoxide dismutase [Fe], Transcriptional regulator, *AraC* family, Heterodimeric efflux ABC transporter, 3-oxoacyl-[acyl-carrier protein] reductase, Transcriptional regulator, *HxlR* family, Porin, Thioredoxin, type 12 methyltransferase, Alpha-acetolactate decarboxylase, Rhodanese-like domain protein, MBL-fold metallo-hydrolase superfamily, Transcriptional regulator, Crp/Fnr family, ThiF family protein, ubiquitin-activating enzyme, Mycobacteriophage Barnyard protein *gp56*
GR7	4,080,427	4,134,730	54,304	Phage endolysin, C-5 cytosine-specific DNA methylase, ATP-dependent RNA helicase *YejH*

Integrative and conjugative elements (ICEs) are relatively common among *E. anophelis* genomes and the Wisconsin *E. anophelis* strain had it disrupted *mutY* gene due to insertion of an ICE*Ea*I. Therefore, we surveyed the presence of ICEs among the genomes of our isolates. The characteristic genes associated with ICE*Ea*III and ICE*Ea*I were found in GR1 and GR6, respectively. Despite an ICE*Ea*I was found in our specific lineage, the locus of insertion was different from that of the Wisconsin strain, and the *mutY* gene was intact in our strains. Besides, the cargo genes carried by the ICE*Ea*I were also very different in compared to those in the Wisconsin strain (Fig. S2). The presence and insertion locations of different ICEs of our strains and other outbreak representative strains are illustrated in Fig. S1.

### Primer design and prevalence screening for outbreak strain.

**FIG 4:**
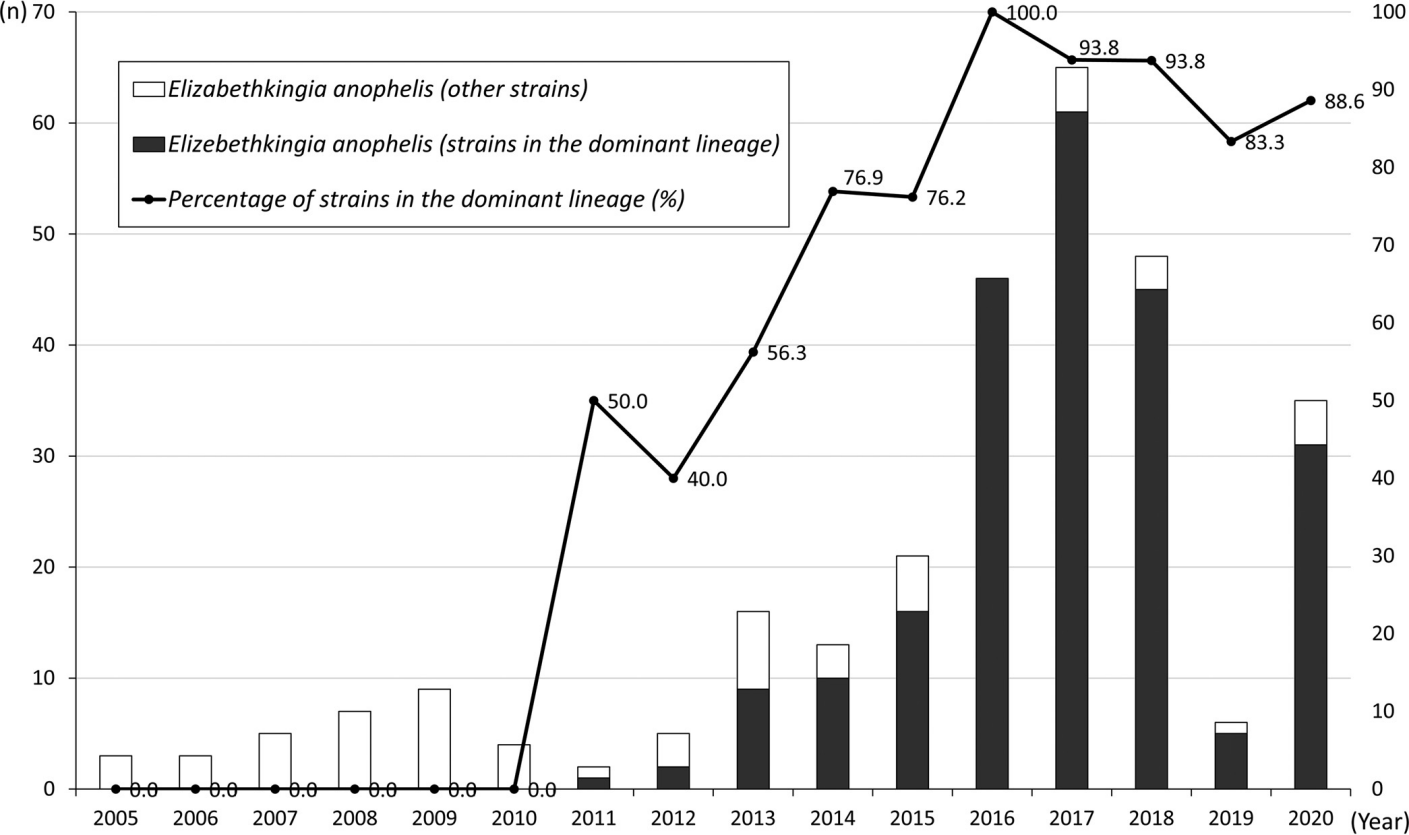
The annual number of Elizabethkingia anophelis strains in the Changhua Christian Hospital from 2005 to 2020 and the percentage of *E. anophelis* strains belonging to the dominant lineage.

The primers designed for multiplex PCR are listed in Table 2 including three primer pairs were designed specifically for target sequences within GR3, GR4 and GR5. We omitted GR1 and GR6 because these are likely ICEs, which are potentially mobile and not ideal targets. GR2 was not used because it was also present in strain 277-17, a strain not belonging to the dominant lineage by PFGE analysis. To ensure the specificity of the three primer pairs, we had avoided sequences from any mobilized elements such as phages, transposons, integrons and repetitive sequences. In addition, the primers were also checked using NCBI database and their specificity were confirmed. The result of a rapid screen by multiplex PCR revealed that the *E. anophelis* strain with genomic features as the dominant lineage was first detected in 2011 and the case number of those specific strains increased significantly from 2011 to 2020 (Fig. 4). We also investigated 90 isolates from multiple hospitals in the TSAR program between 2002 and 2018 (18). The earliest isolate belonging to the dominant lineage was identified in 2008 from a hospital in northern Taiwan. The ratio of strains in the specific *E. anophelis* lineage also increased from 6.25% (3/48) during 2002–2012 to 33.3% (14/42) during 2014–2018; of note, it was 50% (*n* = 7/14) in 2018 (Fig. S3).

**TABLE 2:** Primers designed for multiplex PCR from specific genomic regions (GRs) in the study to evaluate the prevalence of dominant Elizabethkingia anophelis strains

Region	Primer name	Sequence (5′–3′)	Size (mer.)	*T_m_* (°C)	GC (%)	Product size (bp)	Target gene
GR3	Ez_R3-1F	gAggCAAATTggAAAgAgT	19	46.8	42	1026	From the CMP/dCMP deaminase gene to the zinc binding domain-containing protein gene
GR3	Ez_R3-1R	TTCTgggTAAgTTggTgT	18	45.8	44		
GR4	Ez_R4-1F	TATTgTgAgCCCTTCgTT	18	45.8	44	681	Type II, N-methyl DNA methyltransferase gene
GR4	Ez_R4-1R	CATTTCCgTCTTggTCTT	18	45.8	44		
GR5	Ez_R5-1F	CgggACATAACgCAAATA	18	45.8	44	249	N-acetylmuramonyl-l-alanine amidase gene
GR5	Ez_R5-1R	gCCAgTTTCTAACATCgAA	19	46.8	42		

### Antimicrobial susceptibility and the clinical features between patients infected by different *E. anophelis* strains.

The antimicrobial susceptibility between strains belonging to the dominant lineage and others were compared (Table 3). All *E. anophelis* strains were resistant to most of the antimicrobial agents tested. The susceptible rates of trimethoprim-sulfamethoxazole were about 50–60% without statistically significant difference noted between two groups. The most prominent difference was the susceptibility to ciprofloxacin; all strains in the dominant lineage were resistant to ciprofloxacin (100%, 226/226) which was higher than other strains (27.4%, 17/62). All our 18 sequenced strains in the dominant lineage had DNA gyrase subunit A (*gyrA*) mutations at codons 83, Ser83Ile, that was associated with ciprofloxacin resistance. Instead, 3 other *E. anophelis* strains did not have the *gyrA* mutation (strain 313-22, 2-6 and 2–14) and another strain 277-17 carried a single mutation, Ser83Arg. All 22 sequenced *E. anophelis* isolates carried multiple β-lactamases genes (*bla*_CME-1_ and *bla*_GOB_) which were associated extensive drug resistance to β-lactams. The strain 277-17 also carried genes conferring resistance to macrolides (*ere*(D), *erm*(F)), sulfonamide (*sul2*) and tetracycline (*tet[X]*) but those genes were not found among 18 sequenced strains in the dominant lineage. The MIC of minocycline determined by E-tests ranged from 0.19 to 0.5 μg/ml among 18 *E. anophelis* strains in our dominant lineage, which were considered susceptible (≦ 4 μg/ml) by using the breakpoints for other non-*Enterobacterales* according to CLSI M100-S30 criteria.

**TABLE 3:** Antimicrobial MIC and susceptibility of Elizabethkingia anophelis strains in Changhua Christian Hospital^*a*^

	*E. anophelis* (strains of dominant lineage, *n* = 226)			*E. anophelis* (other strains, *n* = 62)		
Characteristics	MIC_50_ (μg/mL)	MIC_90_ (μg/mL)	S, n (%)	MIC_50_ (μg/mL)	MIC_90_ (μg/mL)	S, n (%)
Amikacin	≥ 64	≥ 64	0 (0)	≥ 64	≥ 64	3 (4.8)
Cefepime	≥ 64	≥ 64	0 (0)	≥ 64	≥ 64	13 (19.7)
Cefotaxime	≥ 64	≥ 64	0 (0)	≥ 64	≥ 64	0 (0)
Ceftazidime	≥ 32	≥ 32	0 (0)	≥ 32	≥ 32	0 (0)
Ciprofloxacin	≥ 4	≥ 4	0 (0)	2	≥ 4	45 (72.6)^*b*^
Colistin	≥ 16	≥ 16	0 (0)	≥ 16	≥ 16	0 (0)
Flomoxef	≥ 64	≥ 64	0 (0)	≥ 64	≥ 64	0 (0)
Gentamicin	≥ 16	≥ 16	1 (0.4)	≥ 16	≥ 16	1 (1.6)
Imipenem	≥ 16	≥ 16	0 (0)	≥ 16	≥ 16	0 (0)
Piperacillin-tazobactam	≥ 128/4	≥ 128/4	0 (0)	≥ 128/4	≥ 128/4	0 (0)
Trimethoprim-sulfamethoxazole	≤ 2/38	≥ 4/76	133 (58.8)	≤ 2/38	≥ 4/76	36 (58.1)

a S, susceptible; MIC50, MIC at which 50% of the isolates tested are inhibited; MIC90, MIC at which 90% of the isolates tested are inhibited; n (%), the number of susceptible isolates and susceptible rates.

b *P* <0.05; comparison for the susceptible rates between dominant *E. anophelis* strains to other *E. anophelis* strains

For 288 patients with bacteremia due to *E. anophelis*, 226 (78.5%) were infected by *E. anophelis* strains belonging to the dominant lineage and 62 (21.5%) were by other strains (Table 4). The average age and sex distribution were similar between two groups. Strains in the dominant lineage had higher probability to be isolated from patients admitted to respiratory care centers or respiratory wards; therefore, more patients in this group required mechanical ventilators supports (78.3% [177/226] versus 46.8%, [29/62], *P* < 0.001). Most patients were treated with trimethoprim-sulfamethoxazole. Fluoroquinolones were less used for patients infected by strains in the dominant lineage because of high resistance rates; instead, more patients received minocycline as alternative treatment (Fig. 4). Besides, glycopeptide (vancomycin) and penicillin (piperacillin) were more commonly used for patients infected by other strains in early period, which was probably attributable to former susceptibility reports determined by disk methods showing inhibitory zones for vancomycin and piperacillin before 2010. Finally, the mortality rates were higher among patients infected by strains in the dominant lineage than those by other strains both in 14-day mortality rates (33.2%, 75/226 versus 16.1%, 10/62, *P* = 0.009) and in-hospital mortality rates (50.9%, 115/226 versus 32.2%, 20/62, *P* = 0.014).

**TABLE 4:** Demographic variables associated with Elizabethkingia anophelis bacteremia by strains belonging to the specific lineage and other strains in Changhua Christian Hospital^*a*^

Variable	*E. anophelis* strains of dominant lineage (*n* = 226)	Other *E. anophelis* strains (*n* = 62)	*P* value
Age	73.4	71.9	0.456
Sex, male, *n* (%)	135 (59.7)	40 (64.5)	0.495
Duration of admission, median (IQR), days	56 (35.0–84.0)	44 (28.3–106.7)	0.057
Ward, *n* (%)			0.035
ICU	79 (35.0)	29 (46.8)	
RCC	53 (23.5)	6 (9.7)	
RCW	46 (20.4)	11 (17.7)	
Ward	48 (21.2)	15 (24.2)	
OPD	0 (0)	1 (1.6)	
Period of admission, *n* (%)			
2005−2010	0 (0)	31 (50.0)	< 0.001
2011−2015	38 (16.8)	19 (30.6)	
2016−2020	188 (83.2)	12 (19.4)	
Charlson comorbidity index, median (IQR)	3 (2.0–4.0)	3 (2.0–6.0)	0.0939
Comorbidities, *n* (%)^*b*^			
Solid-organ tumor	35 (15.9)	15 (24.2)	0.132
Diabetes mellitus	91 (40.3)	28 (45.2)	0.489
Chronic pulmonary disease	41 (18.1)	12 (19.4)	0.827
Chronic kidney disease	73 (32.3)	18 (29.0)	0.624
Hematologic malignancy	8 (3.5)	6 (9.7)	0.047
Dementia	24 (10.6)	12 (19.4)	0.066
Connective tissue disease	8 (3.5)	1 (1.6)	0.441
Chronic liver disease	32 (14.2)	13 (21.0)	0.192
Steroid use	17 (7.5)	4 (6.5)	0.774
Mechanical ventilation	177 (78.3)	29 (46.8)	<0.001
Antimicrobial treatment, *n* (%)^*b*^			
No treatment	27 (11.9)	3 (4.8)	0.105
Aminoglycoside	3 (1.3)	2 (3.2)	0.312
Carbapenem	12 (5.3)	4 (6.5)	0.729
Cephalosporin	13 (5.8)	7 (11.3)	0.129
Glycopeptide	34 (15.0)	16 (25.8)	0.048
Fluoroquinolone	24 (10.6)	20 (32.3)	<0.001
Minocycline	37 (16.4)	1 (1.6)	0.002
Penicillin^*c*^	20 (8.8)	13 (21.0)	0.008
Trimethoprim/sulfamethoxazole	149 (65.9)	43 (69.4)	0.613
Mortality, *n* (%)			
14-day mortality	75 (33.2)	10 (16.1)	0.009
In hospital mortality	115 (50.9)	20 (32.2)	0.014

a ICU, intensive care unit; RCC, respiratory care center; RCW, respiratory care ward; OPD, outpatient department.

b May be multiple.

c Includes ampicillin/sulbactam, piperacillin, piperacillin-tazobactam.

## DISCUSSION

In this study, we identified a specific lineage of *E. anophelis* strains that evolved and emerged in recent decades in Taiwan. Through whole-genome sequencing and comparative genomic analysis, the specific *E. anophelis* lineage were proved different from any currently known isolates around the world and associated with extensive antimicrobial resistance and high mortality rates.

A recent study explored the relatedness of 22 indigenous *E. anophelis* in Australia over a period of 16 years. Some *E. anophelis* isolates in Australia are genetically related to the strains from the Unites States, England, Singapore and Taiwan, which implies international spread of *E. anophelis* isolates (15). From 2015 to 2016, a unique *E. anophelis* strain was reported in a large-scale outbreak in WI, USA (7, 10). Similarly, a specific lineage of *E. anophelis* strains was identified in our study with unique genomic features (20). Although cg-MLST genotyping discriminated our *E. anophelis* strains from the others well, it has not escaped our notice that, strain 277-17, which does not belong to the dominant lineage by PFGE analysis was clustered together with the 18 dominant lineage stains in the cg-MLST tree (22). It is the comparative genomics analysis on the presence of the GRs revealed that *E. anophelis* 277-17 had quite different genomic features from those strains in the dominant lineage. As lineage-specific accessories carried in the genomes, some of these GRs are actually good markers for discriminating the dominant lineage.

In our study, all *E. anophelis* strains in the specific lineage were resistant to ciprofloxacin due to the *gyrA* mutation, Ser83Ile. A previous study reported 12.5% (9/72) of *E. anophelis* isolates from a university-affiliated hospital in southern Taiwan between 2005 and 2018 had Ser83Ile mutation in gyrA (23). In 2020, Wang et al. reported fluoroquinolone resistance among 34.4% (11/32) of *E. anophelis* isolates and 9 had amino acid substitutions Ser83Ile in Shanghai, China (24). A clonal expansion of our strains may lead to increasing resistance to ciprofloxacin. On the other hand, all 18 sequenced strains in the dominant lineage were lack of *tet*(X) gene that associated with tetracycline resistance. The MICs of 18 strains were all less than 4 μg/ml. The result was similar to those of many recent studies (24–26). A study in Shanghai, China evaluated antimicrobial susceptibility of 52 *Elizabethkingia* isolates (including 35 *E. anophelis*, 14 *E. meningoseptica*, and 2 *E. miricola*) and minocycline was more active than doxycycline and tigecycline (≤ 4 μg/ml, 100% versus 96.2% and 78.8%, respectively) (24). Another study in Taiwan also demonstrated that 100% (90/90), 96.7% (87/90), and 52.2% (47/90) of *E. anophelis* isolates were susceptible to minocycline, doxycycline and tigecycline, respectively (18). Though no current breakpoint value is available, the clinical use of tetracycline, especially for minocycline and doxycycline, to treat *E. anophelis* infections warrants more investigations.

Several gene clusters related to membrane-associated transporter functions were identified in the GRs specific to the specific lineage of *E. anophelis* strains; these included genes for an MFS-type transporter in GR2, genes for an RND-type efflux system in GR4, gene clusters for two ABC-type transporter systems in GR6, genes for outer membrane receptors and several genes for porins. The MFS, RND, and ABC-type transporters have been long regarded as members of the bacterial efflux pump systems that may play a role in facilitating the efflux transport of antimicrobials, ions, or bioactive molecules across the membrane (27, 28). In a recent review, the bacterial efflux pump is not only associated with antibiotic resistance but also play roles in quorum sensing, biofilm formation, pathogenicity and virulence (29, 30). It is plausible that these extra transporter genes in the GRs may be a benefit to the dominant of the specific lineage of *E. anophelis* strains.

There are several limitations in this study. First, molecular epidemiologic studies could be extended to investigate the local and global trends of *E. anophelis* infections related to the specific lineage with time. Second, while some antimicrobial agents such as vancomycin or rifampin were reported to be active *in vitro* against some *Elizabethkingia* species (18, 31), we did not assess the impact of antimicrobial therapy chosen based on the available antimicrobial susceptibility on the outcome of *E. anophelis* infections.

In conclusion, our study revealed an increasing trend of infection due to a specific lineage of *E. anophelis* strains. Those specific strains were associated with extensive antimicrobial resistance and high mortality rates. The genomic features identified would be helpful for further evaluation of the evolving epidemiology, antimicrobial resistance, virulence and modes of transmission.

## MATERIALS AND METHODS

### Setting and bacterial isolates.

All nonduplicated *Elizabethkingia* species isolated from patients between 2005 and 2020 were retrieved retrospectively from the biobank at Changhua Christian Hospital (CCH). All isolates were reidentified by the Matrix Assisted Laser Desorption Ionization Time-of-Flight (MALDI-TOF) technology, VitekMS with Knowledge Base Version 3.0 (bioMérieux SA, Marcy L’Étoile, France) as previously reported (17, 20). The antimicrobial susceptibility was determined using Vitek 2 system and susceptibility testing cards (bioMérieux, Lyon, France). The MIC of minocycline was determined by Etest (bioMérieux, Marcy l'Etoile, France) according to the manufacturer's recommendations (32). The breakpoints for antimicrobial susceptibility was interpreted using criteria for other non-*Enterobacterales* according to CLSI M100-S30 (33). The breakpoint used for colistin was adapted from the criteria for Pseudomonas aeruginosa that a MIC ≥ 4 μg/ml is categorized as resistance in the CLSI M100-S30 (33). A standardized case record form was used to collect information on patients with *E. anophelis* bacteremia.

The *E. anophelis* isolates collected in the Taiwan Surveillance of Antimicrobial Resistance (TSAR) program from 2002 (TSARIII) to 2018 (TSARIX) were included in trend analysis. The TSAR program is a biannual nationwide bacteria repository program in Taiwan since 1998 (18).

### Whole-genome sequencing, core-genome multilocus sequence typing, and comparative genomic analysis of *E. anophelis* strains.

Whole-genome sequencing were performed using Illumina iSeq100 for short-read sequencing and Nanopore MinION platforms for long-read sequencing as reported previously (34). Briefly, the libraries of short-read sequencing were constructed using the Nextera DNA CD Indexes (Illumina) and analyzed by Agilent 2100 Bioanalyzer that generated 150 bp paired end reads, followed by adaptor trimming. For long-read sequencing, libraries were constructed using Rapid Barcoding kit SQK-RBK004 (Oxford Nanopore Technologies) and sequenced on a Nanopore MinION following standard protocols (34). Hybrid assembly of the Illumina short paired-end reads and Nanopore long reads were performed using Unicycler version 0.4.8 to result in a single circular chromosome of all isolates (4). The correctness of the assembly results was rechecked by using another assembler, Canu 1.8, with the aid of CLC genomic workbench 11 (Qiagen). The genomes were annotated by the NCBI Prokaryotic Genome Annotation Pipeline (PGAP). The core-genome multilocus sequence typing (cg-MLST) phylogenetic tree analysis was conducted using Harvest suite, Parsnp v1.2 module with default settings (35). The phylogenetic result was visualized using CLC genomic workbench v11.0.11 (Qiagen).

Comparative genomic analyses were performed using blast, MAUVE v1.0 and BRIG 0.95 to determine strain-specific genomic regions (36, 37). Antimicrobial resistance gene were predicted using Resfinder v3.2 (38). Three types of integrative and conjugative elements (ICEs) were determined according to the architecture of the conjugation module and the phylogeny of the relaxase, coupling protein, TraG, and TraJ protein sequences as reported previously (35). Islandviewer4 with two genomic island prediction tools (SIGI-HMM and IslandPath-DIMOB) was used to predict genomic islands (39). Phage genomes were analyzed by PHAST (40). With reference to the result of comparative genomic analysis, we designed primer pairs in CLC genomic workbench 11 for rapid epidemiological survey. Oligomers were synthesized by Tri-I Biotech Inc. (Taichung city, Taiwan).

### Ethics and experimental biosafety statements.

This study was approved by the Institutional Review Board of Changhua Christian Hospital (registration no. 180502). Informed consent was waived. The experiments were also approved by the Institutional Biosafety Committee of Changhua Christian Hospital.

### Statistical analysis.

MedCalc software (MedCalc Software Ltd., Los Angeles, CA, USA) was used for statistical analyses. Chi-square or Fisher’s exact tests were used to compare categorical variables. Noncategorical variables were compared by Student's *t* test for data with normal distribution or by the Mann-Whitney U-test for data without normal distribution. Trend analysis was performed by Cochran-Armitage test. All tests were two-tailed and a *P value* of <0.05 was considered significant.

### Data availability.

The complete genome sequencing data of *E. anophelis* strains in this study have been deposited at GenBank under the accession numbers CP046080, CP071530, CP071531, CP071532, CP071533, CP071534, CP071535, CP0716, CP071537, CP071538, CP071539, CP071540, CP071541, CP071542, CP071543, CP071544, CP071545, CP071546, CP071547, CP071548, CP071550, CP07155for *E. anophelis* 296-96, 2-8, 367-19, 362-2, 356-17, 355-58, 354-87, 354-72, 351-72, 349-83, 348-50, 347-96, 347-50, 347-10, 346-72, 346-66, 344-1, 343-63, 313-22, 277-17, 2-14, and 2-62, respectively. The BioProject numbers included PRJNA589371 and PRJNA707236.
