# Supplementary material for: The Evolutionary Trend and Genomic Features of an Emerging Lineage of Elizabethkingia anophelis Strains in Taiwan
Source: Microbiol Spectr. 2022 Jan 19;10(1):e01682-21. doi: 10.1128/spectrum.01682-21 (PMC8768576; doi:10.1128/spectrum.01682-21)
Supplement: SUPPLEMENTAL FILE 1 — Supplemental material. Download SPECTRUM01682-21_Supp_1_seq13.pdf, PDF file, 0.5 MB [file spectrum01682-21_supp_1_seq13.pdf]

Table S1. The *Elizabethkingia anophelis* isolates with available genome data included in the core-genome multi-locus sequence typing

Strain	source	Region	Level	Size (Mb)	GC (%)	Accession number	Scaffolds	Genes	Proteins (CDS)
R26	Mosquito	Stockholm, Sweden	Complete Genome	4.06	35.5	chromosome: NZ_CP023401.1/C 3401.1	1	3741	3635
DSM-23781		Justus Liebig University, Germany	Contig	4.07	35.4	FLST01	58	3779	3675
As1	Mosquito	US	Contig	3.59	35.5	LFKT01	12	3315	3237
AR4-6	Mosquito	Sichuan, China	Complete Genome	4.09	35.5	chromosome: NZ_CP023404.1/ CP023404.1	1	3785	3678
AR6-8	Mosquito	Sichuan, China	Complete Genome	4.09	35.5	chromosome: NZ_CP023403.1/ CP023403.1	1	3785	3678
277-17	Human patient	Taiwan	Complete Genome	4.00	35.6	CP071549	2		3842

346-72	Human patient	Taiwan	Complete Genome	4.10	35.8	CP071543	1		3960
347-96	Human patient	Taiwan	Complete Genome	4.10	35.8	CP071540	1		3965
354-72	Human patient	Taiwan	Complete Genome	4.10	35.8	CP071536	1		3963
354-87	Human patient	Taiwan	Complete Genome	4.10	35.9	CP071535	1		3926
355-58	Human patient	Taiwan	Complete Genome	4.10	35.8	CP071534	1		3963
356-17	Human patient	Taiwan	Complete Genome	4.10	35.8	CP071533	1		3961
346-66	Human patient	Taiwan	Complete Genome	4.10	35.8	CP071544	1		3966
347-10	Human patient	Taiwan	Complete Genome	4.10	35.8	CP071542	1		3962
343-63	Human patient	Taiwan	Complete Genome	4.10	35.8	CP071546	1		3951
347-50	Human patient	Taiwan	Complete Genome	4.10	35.9	CP071541	1		3925
348-50	Human patient	Taiwan	Complete Genome	4.10	35.8	CP071539	1		3961

349-83	Human patient	Taiwan	Complete Genome	4.10	35.8	CP071538	1		3967
351-72	Human patient	Taiwan	Complete Genome	4.10	35.8	CP071537	1		3964
362-2	Human patient	Taiwan	Complete Genome	4.10	35.8	CP071532	1		3962
367-19	Human patient	Taiwan	Complete Genome	4.10	35.8	CP071531	1		3967
296-96	Human patient	Taiwan	Complete Genome	4.20	35.8	chromosome:NZ_CP046080.1/CP046080.1	1	3898	3779
344-1	Human patient	Taiwan	Complete Genome	4.10	35.9	CP071545	2		4017
2-62	Suction Rotary knob	Taiwan	Complete Genome	4.10	35.8	CP071551	1		3982
GX130	Human patient	Guangxi Zhuang Autonomous Region, China	Contig	3.87	35.6	SYWB01	38	3543	3452
313-22	Human patient	Taiwan	Complete Genome	4.00	35.8	CP071547	1		3799

CSID_3015183678	Human patient	Wisconsin, US	Complete Genome	3.93	35.8	chromosome:NZ_CP014805.2/CP014805.2	1	3587	3483
CSID_3015183684	Human patient	Wisconsin, US	Complete Genome	3.93	35.8	chromosome:NZ_CP015066.2/CP015066.2	1	3587	3481
CSID_3000521207	Human patient	Wisconsin, US	Complete Genome	3.85	35.7	chromosome:NZ_CP015067.2/CP015067.2	1	3513	3412
CSID_3015183681	Human patient	Wisconsin, US	Complete Genome	3.93	35.8	chromosome:NZ_CP015068.2/CP015068.2	1	3586	3482
FDAARGOS_132	Human patient	Washington, US	Complete Genome	4.00	35.7	chromosome:NZ_CP014020.1/CP014020.1	1	3675	3565
FDAARGOS_134	Human patient	Washington, US	Complete Genome	4.00	35.7	chromosome:NZ_CP014021.1/CP014021.1	1	3676	3567
PHOL-090	Human patient	Greater Toronto Area, Canada	Contig	4.00	35.5	RSAZ01	27	3679	3556

F3543	Human patient	Flordia, US	Complete Genome	3.97	35.6	chromosome:NZ_CP014340.1/CP014340.1	1	3638	3522
3375	Human patient	South California, US	Complete Genome	4.01	35.7	chromosome:NZ_CP016373.1/CP016373.1	1	3704	3583
FDAARGOS_198	Human patient	Sweden	Complete Genome	4.07	35.8	chromosome:NZ_CP023010.2/CP023010.2	1	3737	3614
BP8467	Human patient	Vellore, India	Contig	3.86	35.6	VTFI01	30	3527	3394
SP4182	Human patient	Vellore, India	Contig	3.83	35.5	PXJH01	47	3619	873
40313151	Human patient	US	Contig	4.10	35.6	QNTY01	18	3833	3735
E6809	Human patient	California, US	Complete Genome	4.12	35.6	chromosome:NZ_CP014339.1/CP014339.1	1	3821	3703
6499925	Human patient	US	Contig	3.96	35.8	QNTZ01	16	3641	3538
CSID_3000516074	Human patient	Illinois, US	Contig	4.05	35.7	MAHA01	17	3744	3639

CIP111067		Institut Pasteur, Paris	Scaffold	3.85	35.7	FTQZ01	65	3508	3414
PHOL-104	Human patient	Greater Toronto Area, Canada	Contig	3.84	35.6	RSAW01	43	3497	3400
CSID_3000516810	Human patient	Arkansas, US	Contig	3.87	35.6	MAHH01	25	3530	3440
Ea_2-8	Tap water	Taiwan	Complete Genome	4.00	35.6	CP071530	1		3751
PW2806		European Bioinformatics Institute, UK	Scaffold	3.91	35.9	CBYD01	42	3616	3494
PW2809		European Bioinformatics Institute, UK	Scaffold	3.92	35.8	CBYE01	32	3623	3499

Ea_0422	Human patient	Flordia, US	Complete Genome	3.99	35.6	chromosome:NZ_CP016370.1/CP016370.1	1	3698	3585
CIP60.58		Institut Pasteur, Paris	Contig	3.96	35.6	FTQY01	24	3679	3581
CSID_3015183679	Human patient	Wisconsin, US	Contig	3.84	35.6	MAHO01	23	3567	3472
CSID_3015183680	Human patient	Wisconsin, US	Contig	4.00	35.5	MAHP01	38	3711	3605
JUNP 353	Human patient	Kathmandu, Nepal	Complete Genome	4.32	35.8	chromosome:NZ_AP022313.1/AP022313.1	1	4049	3897
NUH4	hand hygiene sink aerator of the neonatal ICU ward of National University Hospital	Singapore	Contig	4.24	35.6	ASYI01	50	3943	3831

NUHP1	Human patient	Singapore	Complete Genome	4.37	35.6	chromosome:NZ_CP007547.1/CP007547.1	1	4040	3915
NUHP3	Human patient	Singapore	Contig	4.33	35.5	ASYG01	71	4026	3909
NUH1	hand hygiene sink aerator	Singapore	Contig	4.33	35.6	ASYH01	59	4026	3908
NUHP2	Human patient	Singapore	Contig	4.33	35.5	ASYF01	59	4026	3915
12012-2 PRCM	Human patient	Fujian Quanzhou Hospital, China	Contig	4.02	35.6	LPXG01	83	3711	3595
LDVH-AR107	*Cyprinus carpio*	France	Scaffold	3.99	35.7	FTPG01	105	3667	3555
Po0527107		European Bioinformatics Institute, UK	Contig	4.03	35.5	CCAC01	89	3708	3601
V0378064		European Bioinforma	Scaffold	4.04	35.7	CCAB01	213	3735	3627

		tics Institute, UK							
CSID_3015183686	Human patient	Wisconsin, US	Contig	4.20	35.6	MAHR01	49	3908	3809
NUH11	hand hygiene sink aerator	Singapore	Contig	4.09	35.6	ASYK01	59	3791	3676
NUH6	hand hygiene sink aerator	Singapore	Contig	4.12	35.6	ASYJ01	74	3847	3733
EM361-97	Human patient	Kaohsiung, Taiwan	Scaffold	4.08	35.7	LWDS00000000.1	18	3763	3634
FMS-007	Human patient	China	Complete Genome	3.94	35.6	chromosome:NZ_CP006576.1/CP006576.1	1	3611	3501
Ea_2-14	Tap water	Taiwan	Complete Genome	4.00	35.5	CP071550	1		3725
502	Human patient	Birmingham, UK	Contig	3.96	35.5	AVCQ01	21	3642	3537
PHOL-515	Human patient	Greater Toronto Area, Canada	Contig	4.11	35.4	RSAY01	55	3765	3634

PW2810		European Bioinformatics Institute, UK	Scaffold	3.98	35.5	CBYF01	38	3614	3481
CIP111046		Institut Pasteur, Paris	Scaffold	3.96	35.6	FTRB01	41	3619	3522
PHOL-785	Human patient	Greater Toronto Area, Canada	Contig	4.06	35.6	RSAV01	15	3707	3603
CSID_3000516978	Human patient	California, US	Contig	3.99	35.5	MAHJ01	16	3647	3551
CSID_3000517066	Human patient	Texas, US	Contig	3.96	35.6	MAHL01	18	3631	3540
Endophthalmitis	Human patient	Puttaparthi, India	Contig	4.02	35.5	JSAA01	167	3729	2302
Ngousso	*Anopheles coluzzii*	Cameroon	Chromosome	4.24	35.3	chromosome: CP034594.1			

JM-87	Corn zea mays	Alabama, US	Complete Genome	4.18	35.5	chromosome:NZ_CP016372.1/CP016372.1	1	3837	3695
F3201	Human patient	Kuwait	Complete Genome	4.28	35.5	chromosome:NZ_CP016374.1/CP016374.1	2	3927	3797
PHOL-537	Human patient	Greater Toronto Area, Canada	Contig	4.15	35.4	RSAX01	38	3788	3676
B2D		Malaysia	Contig	3.94	35.5	JNCG01	50	3589	3501
OSVUM1	*Equine Stall*	Oklahoma, US	Contig	4.14	35.5	PJMA01	5	3778	3653
OSVUM2	*Equus ferus caballus*	Oklahoma, US	Contig	4.10	35.4	PJLZ01	9	3754	3644
CSID_3000653323	Human patient	Tennessee, US	Contig	4.18	35.4	NWMM01	35	3833	3721
CSID_3000726633	Human patient	Illinois, US	Contig	4.18	35.4	NWMI01	43	3832	3710
CSID_3000726811	Human patient	Minnesota, US	Contig	4.18	35.4	NWMH01	28	3829	3710

Table S2. The genomic island prediction results regarding to the seven genomic regions (GRs) of the *Elizabethkingia* anophelis 296-96 genome

Genomic region	SIGI-HMM (start-end)	IslandPath-DIMOB (start-end
GR1	562,287-566,801	
GR2		
GR3		1,419,188-1,447,202
GR4	1,681,211-1,711,208<br>1,713,464-1,719,154<br>1,729,117-1,736,020	1,677,749-1,712,346
GR5		
GR6	3,690,665-3,695,482<br>3,699,314-3,705,182<br>3,718,986-3,730,938<br>3,750,559-3,757,860<br>3,773,194-3,777,216	3,689,204-3,780,934
GR7	4,126,500-4,131,283	4,105,728-4,113,454

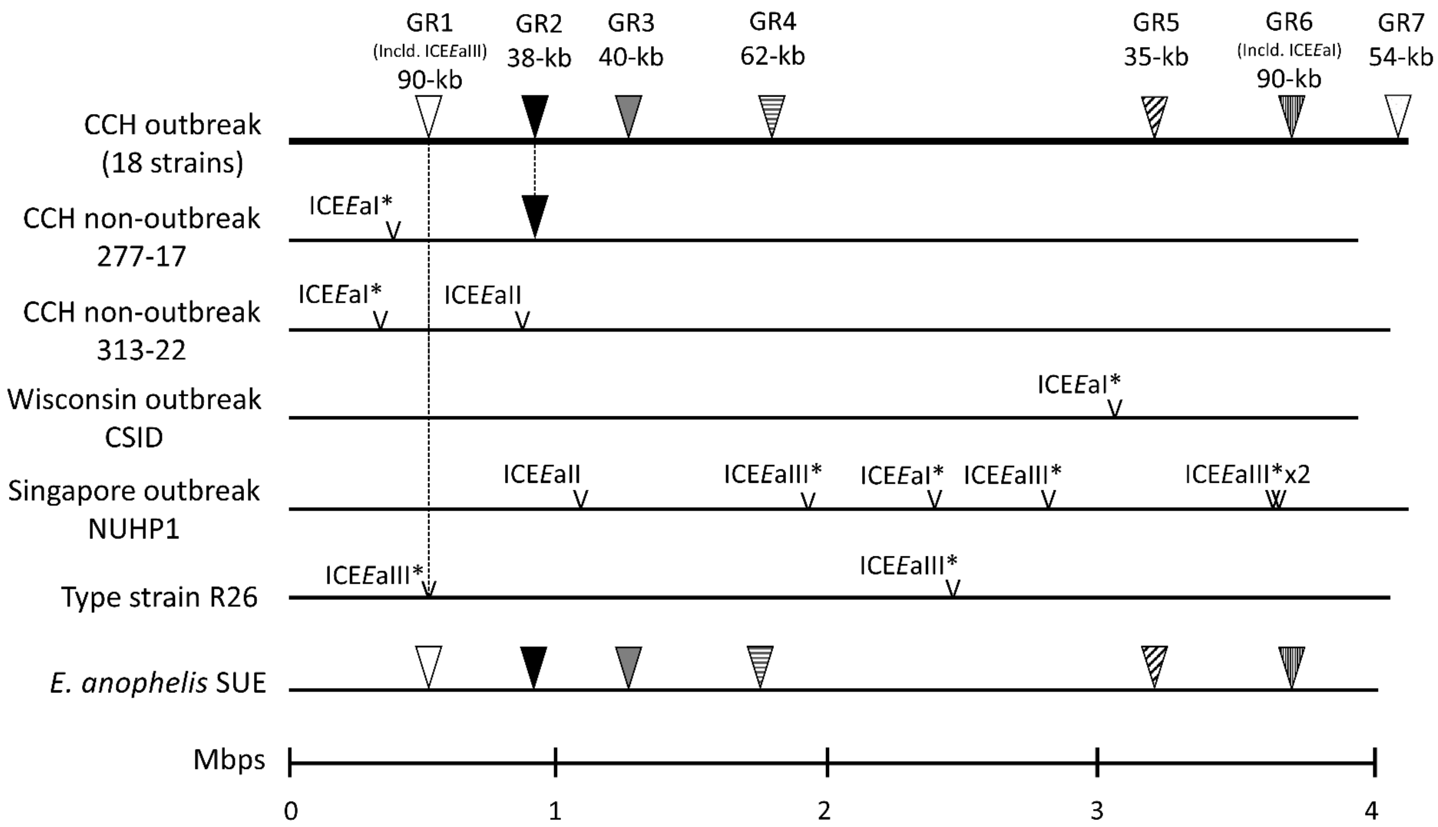

Figure S1. Illustration of seven genomic regions (GRs, triangle) among *Elizabethkingia anophelis* strains including Changhua Christian Hospital (CCH) strains, outbreak strains from Wisconsin and Singapore, R26 and *E. anophelis* SUE. All 18 outbreak

strains had GR1 to GR6 but GR7 only presented in *E. anophelis* 2-62, 344-1 and 296-96. GR1 included integrative and conjugative elements (ICEs) ICE*E*aIII and GR6 included ICE*E*aI. Three types of ICEs were marked in all *E. anophelis* genomes at the locus of insertion (arrow). *ICEs with different cargo gene from the ICEs in the CCH outbreak strains.

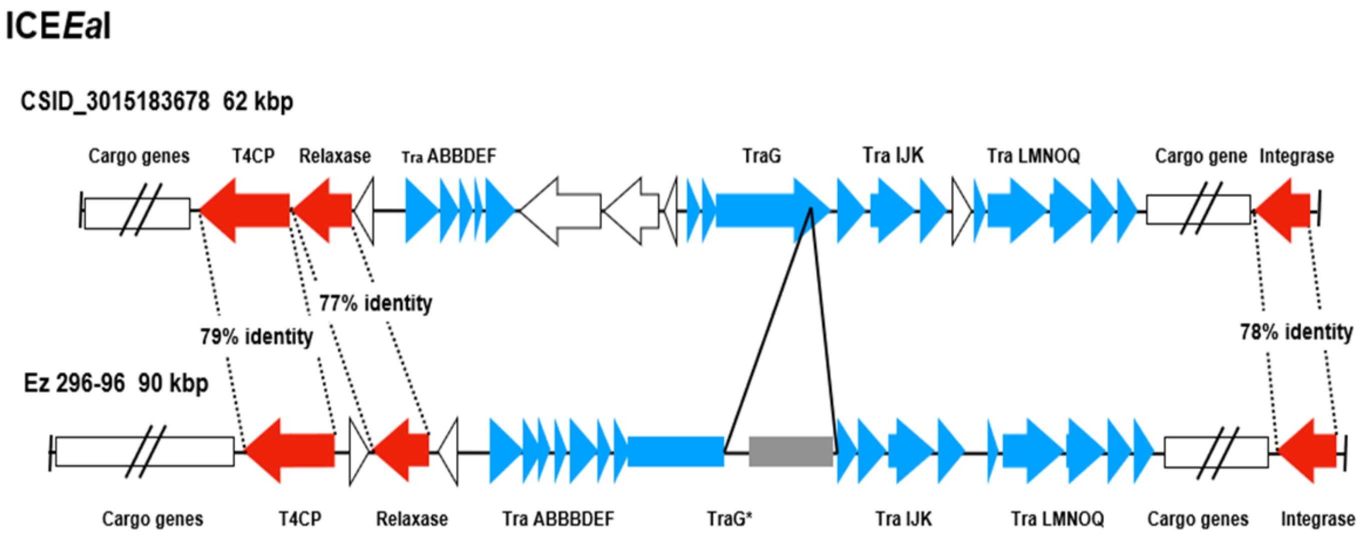

Figure S2. The comparison of the gene structure of integrative and conjugative elements (ICE*Ea*I) between the *Elizabethkingia anophelis* 296-96 in our study and the Wisconsin strain (CSID_3015183678).

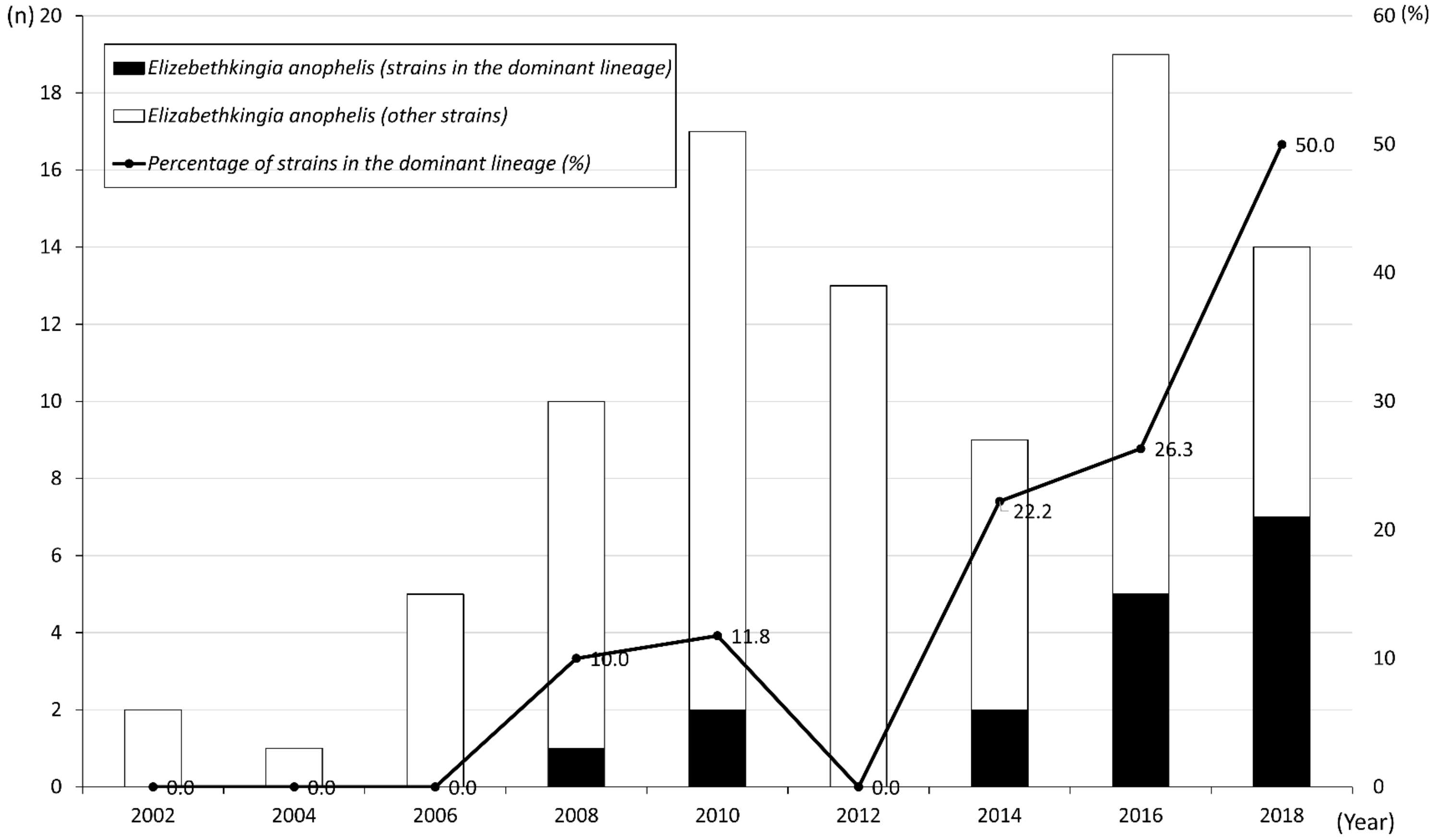

Figure S3. The annual number of *Elizabethkingia anophelis* strains in the Taiwan Surveillance of Antimicrobial Resistance (TSAR) program from 2002 to 2018 and the percentage of *E. anophelis* strains belonging to the dominant lineage
